# Gene looping facilitates TFIIH kinase-mediated termination of transcription

**DOI:** 10.1038/srep12586

**Published:** 2015-08-19

**Authors:** Scott Medler, Athar Ansari

**Affiliations:** 1 Department of Biological Sciences, Wayne State University, 5047 Gullen Mall Detroit, MI 48202

## Abstract

TFIIH is a general transcription factor with kinase and helicase activities. The kinase activity resides in the Kin28 subunit of TFIIH. The role of Kin28 kinase in the early steps of transcription is well established. Here we report a novel role of Kin28 in the termination of transcription. We show that RNAPII reads through a termination signal upon kinase inhibition. Furthermore, the recruitment of termination factors towards the 3′ end of a gene was compromised in the kinase mutant, thus confirming the termination defect. A concomitant decrease in crosslinking of termination factors near the 5′ end of genes was also observed in the kinase-defective mutant. Simultaneous presence of termination factors towards both the ends of a gene is indicative of gene looping; while the loss of termination factor occupancy from the distal ends suggest the abolition of a looped gene conformation. Accordingly, CCC analysis revealed that the looped architecture of genes was severely compromised in the Kin28 kinase mutant. In a looping defective *sua7-1* mutant, even the enzymatically active Kin28 kinase could not rescue the termination defect. These results strongly suggest a crucial role of Kin28 kinase-dependent gene looping in the termination of transcription in budding yeast.

The kinase subunit of TFIIH, Kin28, phosphorylates carboxy-terminal domain (CTD) of RNAPII at serine-5 and serine-7 residues^1^. The Kin28-mediated phosphorylation of serine-5 takes place immediately after initiation and is crucial for promoter clearance^2^,^3^,^4^,^5^. The serine-5 phosphorylation is also required for the recruitment of the capping enzyme for capping of mRNA at the 5′ end^6^,^7^,^8^. The function of serine-7 phosphorylation in transcription of protein coding genes is not clear yet^1^,^9^,^10^

Our initial understanding of the function of TFIIH kinase under physiological conditions has come from studies with the temperature-sensitive mutants of Kin28 in budding yeast. The shifting of Kin28 mutants to the elevated temperature adversely affected the recruitment of TFIIH complex at the promoter resulting in a dramatic decrease in the CTD-serine-5 phosphorylation, and a concomitant decrease in the level of steady state mRNA level^11^,^12^. The temperature-sensitive mutation has been found to affect the catalytic activity of Kin28 kinase as well as its interaction with other subunits of TFIIH^11^,^13^,^14^. To determine the specific role of Kin28 kinase in transcription by RNAPII, the ATP-binding pocket of the enzyme was engineered to make it respond to the specific inhibitor NA-PP1, which is an analog of ATP. In the presence of NA-PP1, the kinase activity of analog-sensitive Kin28 mutant (Kin28-as) is almost completely inhibited without affecting its interaction with the subunits of TFIIH complex^15^. The studies using Kin28-as mutant revealed that the kinase activity is neither required for recruitment of TFIIH at the promoter region, nor it is essential for transcription^5^,^12^,^14^,^16^. A global decrease in the transcript level in the absence of Kin28 kinase activity, however, was observed^12^. This decrease in mRNA level in the Kin28-as mutant was attributed to the effect of serine-5 phosphorylation on the capping of mRNA at the 5′ end rather than a direct role of Kin28 kinase in transcription. This view has been challenged by recent studies, which demonstrated a direct role of Kin28 kinase in transcription^4^,^5^. These studies implicated serine-5 phosphorylation in release of Mediator complex from the promoter-proximal region following initiation of transcription, thereby facilitating promoter clearance.

A number of genes in budding yeast exhibit a physical interaction of the terminator region of the gene with its cognate promoter resulting in the formation of looped gene architecture^17^,^18^,^19^,^20^,^21^,^22^,^23^,^24^,^25^,^26^,^27^,^28^. Formation of such transcription-dependent gene loops has been observed in higher eukaryotes as well^29^,^30^,^31^,^32^,^33^. Gene looping has been implicated in transcription directionality, reinitiation of transcription, transcription memory, intron-mediated transcriptional enhancement and termination of transcription^17^,^19^,^20^,^22^,^23^,^27^,^28^,^29^,^34^. Kin28 has been shown to affect gene looping in budding yeast^24^. It was however not clear in this study if kinase activity of Kin28 is required for gene loop formation. Also, the physiological significance of Kin28-mediated gene looping remained unclear.

To investigate the precise role of Kin28 kinase in transcription by RNAPII, we examined the transcription of a number of inducible and constitutively expressed genes in Kin28-as mutant. Our results suggest that Kin28 kinase is not essential for transcription, but is necessary for the optimal transcription of both inducible and non-inducible genes. Kin28 crosslinked to both the 5′ and 3′ ends of transcriptionally engaged genes. In the absence of kinase activity, localization of Kin28 towards the 3′ end of genes exhibited a dramatic decline. The delocalization of Kin28 from the 3′ end coincided with the polymerase reading through the termination signal with a concomitant loss of looped gene conformation. These results implicate Kin28 kinase activity both in termination of transcription as well as gene looping. We propose that Kin28 kinase-mediated gene looping facilitates termination of transcription in at least a subset of genes in budding yeast.

## Results

### Kin28 kinase is required for optimal transcription of both inducible and constitutively expressed genes

Although CTD-kinase activity of Kin28 is not essential either for transcription or for the survival of yeast cells, growth of cells is severely inhibited in the kinase-defective mutant^5^,^12^,^14^,^15^. These results suggest that either Kin28 kinase is affecting the transcription cycle in a subtle way, or it is playing a role in a yet unknown aspect of cellular dynamic^14^. To further probe the function of Kin28 kinase in transcription by RNAPII, we used the analog-sensitive Kin28-as mutant described earlier^14^,^15^. In the presence of 5–10 μM NA-PP1 analog in the growth medium, the CTD kinase activity of Kin28-as mutant is almost completely inhibited in less than 60 minutes^14^.

To investigate the precise function of Kin28 kinase in transcription cycle, we examined transcription of five inducible (*HXT1*, *MET16*, *CHA1*, *GAL10* and *INO1*) and six constitutively expressed genes (*ACT1*, *ASC1, IMD4*, *MSN5*, *SPC1* and *CMP1*) in Kin28-as mutant in the presence and absence of NA-PP1. The transcription was monitored in terms of steady state mRNA level determined by RT-PCR approach. In the presence of NA-PP1, transcription of both inducible and constitutively expressed genes exhibited a decline by about 2–10 folds in the mutant (Supplemental Figs S1 and S2). No such decrease in RNA level was observed in the isogenic wild type strain in the presence of NA-PP1 or in the Kin28-as strain in the absence of NA-PP1. Our results are in disagreement with another study that did not find a significant decrease in the global mRNA level upon inhibition of Kin28 kinase using the same kin28-as mutant^14^. Using the identical chemical genetic approach, however, another group reported a drastic decrease in mRNA level of a majority of yeast genes upon inhibition of Kin28 kinase^12^. The discrepancy in the result was due to the use of a different normalization control and is discussed in detail in Hong *et al.*^12^. Our results are in agreement with the findings of Hong *et al.*^12^.

**Figure 1:**
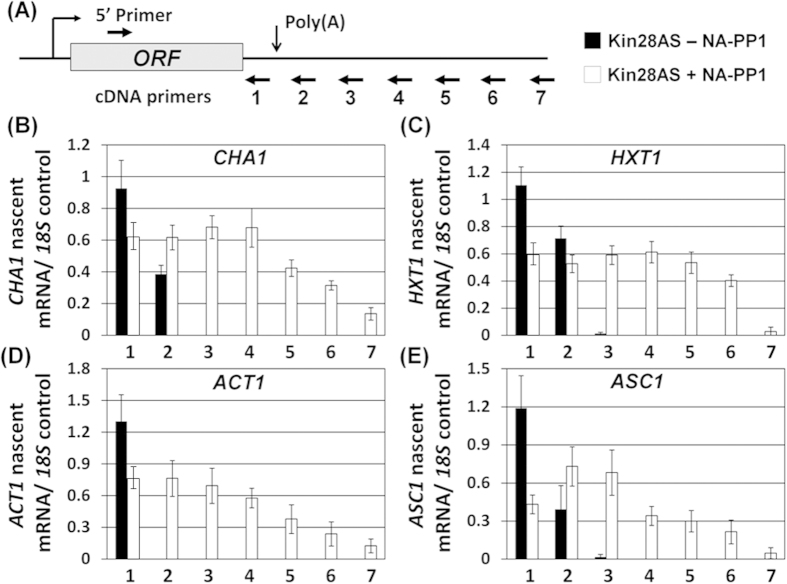
Kin28 kinase inhibition reduces nascent transcription and results in polymerase reading through the termination signal. (**A**) Schematic depiction of a gene showing the positions of three primers 1, 2, 3, 4, 5, 6, 7 and 5′ primer used for cDNA synthesis following TRO procedure. (**B–E**) Quantification of RNA levels detected following TRO analysis in Kin28-as mutant in the absence (-NA-PP1) and presence (+NA-PP1) of NA-PP1. The transcript level of *18S* was used as the control for normalization of results.

The Kin28 kinase activity is essential for capping of mRNA at the 5′ end of genes^35^. Since capping of mRNA has been shown to affect the stability of mRNA, it was possible that the observed decrease in mRNA level of genes in the absence of kinase activity was not due to the effect of kinase on transcription, but on the stability of transcripts. To clarify the issue, we checked the nascent transcription of two inducible genes *CHA1* and *HXT1*, as well as two constitutively expressed genes *ACT1* and *ASC1*, by transcription run-on (TRO) approach. TRO assay is a better indicator of transcriptional activity of a gene than RNAPII density ChIP as it measures nascent transcription of a gene. The Kin28-as cells were grown to the mid-log phase, and transcription was induced in the presence and absence of NA-PP1 as described in Kanin *et al.*^14^. The strand-specific TRO analysis was carried out using Br-UTP by the modification of method described in Core *et al.*, (2008)^34^. The results show that the nascent transcription of *CHA1* and *HXT1* decreased by about 2 fold in the presence of NA-PP1 (Fig. 1B,C). The nascent transcription of two constitutively expressed genes *ACT1* and *ASC1* also registered a steady 3-4 fold decline in the absence of Kin28 kinase activity (Fig. 1D,E). Thus, Kin28 kinase has a direct effect on the transcription of both inducible and non-inducible genes.

### Kin28 kinase is required for termination of transcription

The strand-specific TRO analysis of genes using Br-UTP in the Kin28-as strain revealed a rather unexpected role of Kin28 in RNAPII transcription cycle. In all four genes that we tested, polymerase read through the termination signal in the absence of Kin28 kinase activity thereby implying a defect in transcription termination of these genes (Fig. 1). TRO analysis revealed a very weak or no detectable polymerase signal in the regions 3, 4, 5 and 6 located downstream of the poly(A)-site of *CHA1* and *HXT1* in the mutant in the absence of NA-PP1 (Fig. 1B,C, black bars). In the presence of NA-PP1, however, there was a dramatic increase in TRO signal in the downstream regions 3-6 of both *CHA1* and *HXT1* (Fig. 1B,C, white bars). Similar results were observed for the constitutively expressed genes *ACT1* and *ASC1* (Fig. 1D,E). Thus, RNAPII was not able to read the termination signal of *CHA1*, *HXT1*, *ACT1* and *ASC1* efficiently under kinase-defective condition and continued transcribing the downstream regions. The TRO approach using Br-UTP has been used previously to demonstrate the transcription termination defect in mammalian cells as well^36^,^37^. The downstream TRO signals observed here are not the consequence of transcription of non-coding RNAs like SUTs and CUTs initiating from the vicinity of the poly(A) site as the RNA reverse transcribed using primers 1-7 were PCR amplified using a forward 5′ primer located in the promoter proximal region as shown in Fig. 1A. To corroborate the terminator readthrough phenotype of Kin28-as mutant, we performed RNAPII-density ChIP for *ACT1* and *CHA1* in the presence and absence of NA-PP1. The polymerase readthrough the poly(A) signal of both genes under kinase defective condition thereby affirming the termination defect observed using TRO approach (Supplemental Fig. S3). These results strongly suggest a role for Kin28 kinase in termination of transcription.

**Figure 2:**
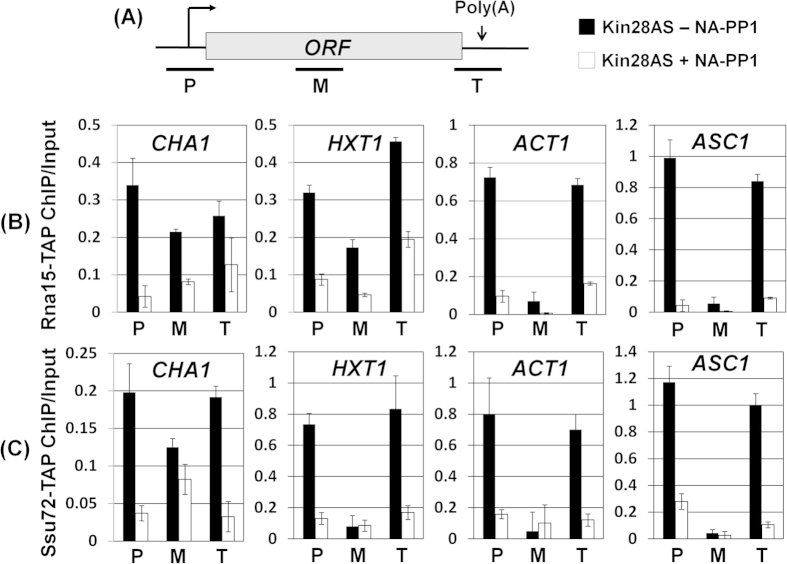
Rna15 and Ssu72 occupancy of the promoter and terminator regions is adversely affected in the absence of Kin28 kinase activity. (**A**) Schematic depiction of a gene indicating the position of the promoter (*P*), coding (*M*) and terminator (*T*) regions amplified by PCR in the ChIP assay. (**B,C**) Quantification of ChIP signals showing crosslinking of Rna15-TAP or Ssu72-TAP to different regions in kin28-as mutant in the absence of NA-PP1 (-NA-PP1) or in the presence of NA-PP1 (+NA-PP1). Input represents DNA prior to immunoprecipitation. Error bars represent one unit of standard deviation.

To further probe the role of Kin28 kinase in termination, we checked the recruitment of CF1 and CPF termination complexes towards the 3′ end of genes in the kinase defective mutant. We expected that if Kin28 kinase activity is required for termination of transcription, the recruitment of either CF1 or CPF complexes may be adversely affected under the kinase defective condition. We used the ChIP approach to monitor the recruitment of CF1 complex towards the 3′ end of genes using its Rna15 subunit, while Ssu72 subunit was used to detect the recruitment of the CPF complex in the mutant strain. ChIP analysis revealed that both Rna15 and Ssu72 occupied the terminator region of all four genes in the mutant in the absence of NA-PP1 (Fig. 2, region T, black bars). In the presence of NA-PP1, however, crosslinking of Rna15 to the 3′ end decreased by about 50-80% (Fig. 2B, region T, white bars), while that of Ssu72 declined by more than 75% (Fig. 2C, region T, white bars). Thus, the recruitment of both CF1 and CPF complexes towards the terminator region of *CHA1*, *HXT1*, *ACT1* and *ASC1* was compromised under the kinase defective condition. Taken together these results strongly suggest a novel role for Kin28 kinase in termination of transcription for at least a subset of genes in budding yeast.

### Kin28 contacts and affects phosphorylation of CTD at the 3′ end of genes

**Figure 3:**
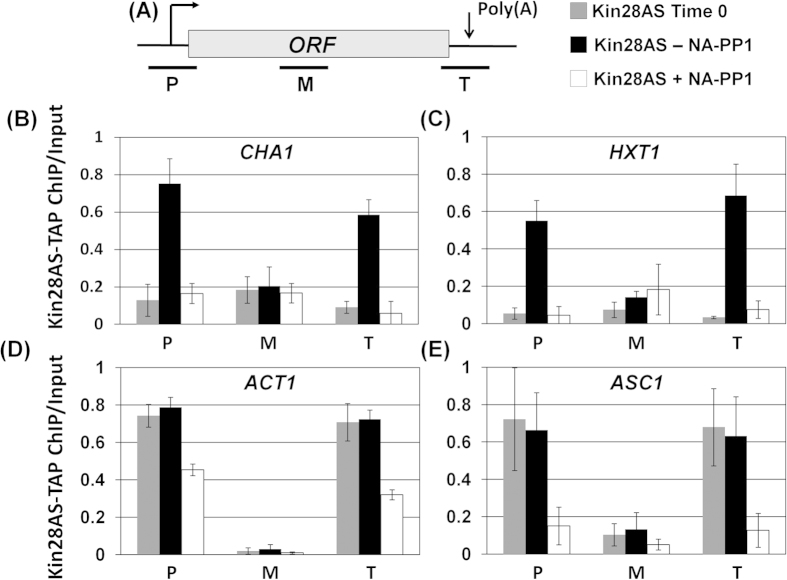
Kin28 occupancy of the promoter and terminator regions is compromised in the absence of Kin28 kinase activity. (**A**) Schematic depiction of a gene indicating the position of the promoter (*P*), coding (*M*) and terminator (*T*) regions PCR amplified in the ChIP assay. (**B–E**) Quantification of ChIP signals showing crosslinking of Kin28 to the promoter, coding and terminator regions of genes in kin28-as mutant in the absence of NA-PP1 (-NA-PP1) or in the presence of NA-PP1 (+NA-PP1). Input represents DNA prior to immunoprecipitation.

The experiments described above firmly established the role of Kin28 kinase in termination of transcription for at least some yeast genes. To have an insight into the role of Kin28 kinase in termination of transcription, we checked if Kin28 brings about termination by physically interacting with the 3′ end of genes and if the kinase activity is required for this interaction. ChIP was performed in a strain bearing TAP-tagged version of Kin28-as allele, in the presence and absence of NA-PP1. As expected, Kin28 was recruited towards the 5′ end of *CHA1*, *HXT1*, *ACT1* and *ASC1* in the absence of NA-PP1 (Fig. 3B–E, region P, black bars). In the presence of NA-PP1, however, crosslinking of Kin28 to the promoter region registered a 50-80% decline (Fig. 3B–E, region P, white bars). Interestingly, Kin28 was also found localized near the 3′ end of genes (Fig. 3B–E, region T, black bars). A genomewide analysis has also found Kin28 crosslinked to the 3′ end of a number of transcriptionally active genes in yeast^38^. It was, however, not clear from this study if the recruitment of Kin28 near the terminator region required its kinase activity. Our results show that the crosslinking of Kin28 towards the terminator region of genes was significantly reduced in the absence of its kinase activity (Fig. 3B–E, region P, white bars). The Kin28 ChIP signal at the 3′ end of *CHA1* and *ACT1* decreased by nearly 50%, while at *HXT1* and *ASC1* a more than 75% decline was observed under kinase-defective condition (Fig. 3B–E). When Kin28 ChIP signal was normalized with the RNAPII signal, we found no net decrease in Kin28 near the 5′ end of genes in the absence of kinase activity. Kin28 signal at the 3′ end, however, still registered a significant decline under kinase-defective condition (Supplemental Fig. S4). These results suggest that the presence of Kin28 at the 3′ end of genes is essential for its termination function.

To confirm the recruitment of Kin28 towards the 3′ end of genes, we examined the phosphorylation status of CTD-serine-5 and CTD-serine-7 near the 3′ end of *CHA1* and *ACT1* in the mutant strain. Although phosphorylation of serine-5 near the promoter-proximal region is well established, there are conflicting reports regarding its phosphorylation near the 3′ end of genes. A few recent studies demonstrated phosphorylation of serine-5 at the 3′ end of a subset of yeast genes^39^,^40^. Our results are in agreement with these reports. We found elevated levels of serine-5 phosphorylation at the 3′ end of both *CHA1* and *ACT1* (Supplemental Figs S5 and S6, region T, black bars). A similar elevated level of serine-7 phosphorylation was observed near the terminator region of both genes (Supplemental Figs S5 and S6, region T, black bars). In the presence of NA-PP1, however, a 2-4 fold decrease in phosphorylation of both serine-5 as well as serine-7 was observed for two genes in the analog-sensitive mutant (Supplemental Figs S5 and S6, region T, white bars). As expected, no appreciable decrease in the level of serine-2 phosphorylation was observed towards the 3′ end of either *CHA1* or *ACT1* in the absence of Kin28 kinase activity (Supplemental Figs S5 and S6, region T).

The overall conclusion of these results is that the recruitment of Kin28 towards the 3′ end of genes as well as Kin28-mediated termination of transcription are both dependent on its kinase activity in budding yeast.

### Kin28 physically interacts with the CF1 and CPF complexes

**Figure 4:**
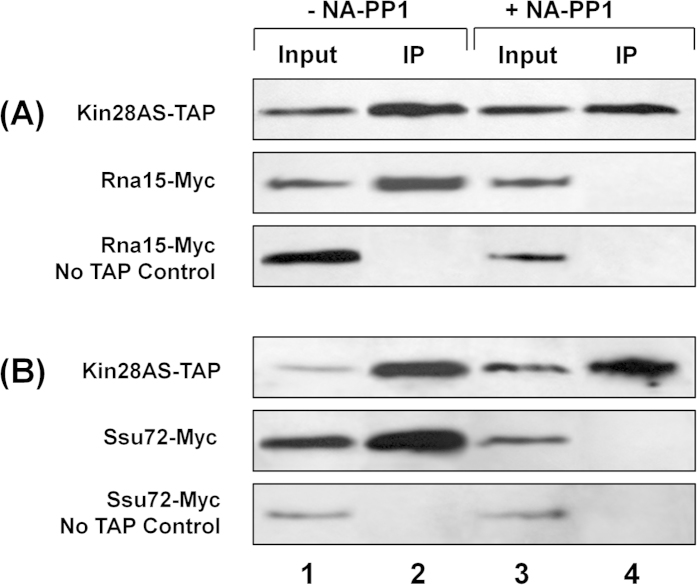
Kin28 physical interacts with Rna15 and Ssu72 in a manner dependent on its kinase activity. (**A**) Western blot analysis showing coimmunoprecipitation of Rna15-Myc with TAP-tagged Kin28 in Kin28-as mutant in the absence (-NA-PP1) and presence (+NA-PP1). (**B**) Western blot analysis showing coimmunoprecipitation of Ssu72-Myc with TAP-tagged Kin28 in Kin28-as mutant in the absence (-NA-PP1) and presence (+NA-PP1). Kin28 was detected by anti-CBP antibodies, while Rna15 and Ssu72 were detected by anti-Myc antibodies. As a control, immunoprecipitation was performed from cells without TAP-tagged Kin28. IP, immunoprecipitation.

The results described above ruled out the possibility of Kin28 kinase playing an indirect role in termination of transcription by affecting CTD-serine-2 phosphorylation. Since inactivation of Kin28 kinase resulted in lowering of both CTD-serine-5 and serine-7 phosphorylation near the 3′ end of genes, the possibility of serine-5 and serine-7 phosphorylation playing a role in transcription termination cannot be ruled out. These results, however, raised the possibility of a direct role of Kin28 in transcription termination by interacting with the CF1 and CPF complexes and facilitating their recruitment towards the 3′ end of genes. We therefore examined the physical interaction of Kin28 with the CF1 and CPF complexes by coimmunoprecipitation approach. The TAP-tagged Kin28 was pulled down using IgG-Sepharose beads, and the presence of either Rna15 or Ssu72 was detected in the immunoprecipitated fraction. Our results show that Kin28 interacts with both CF1 subunit Rna15 and CPF subunit Ssu72 in the absence of NA-PP1 (Fig. 4A,B, lane 2). In the presence of NA-PP1, however, Kin28 interaction with both RNA15 and Ssu72 was completely abolished (Fig. 4A,B, lane 4). These results show that Kin28 interacts with both the CF1 and CPF complexes, and this interaction is dependent on its kinase activity. Whether the Kin28-CF1 and Kin28-CPF interactions are facilitated by the Kin28-mediated phosphorylation of CTD or that of a subunit of CF1 or CPF complex needs further investigation. The possibility of Kin28 influencing termination both indirectly by affecting the CTD-serine-5 phosphorylation in the terminator region, and directly by interacting with the termination factors near the 3′ of the gene also cannot be ruled out.

### Kin28 kinase is required for gene looping

**Figure 5:**
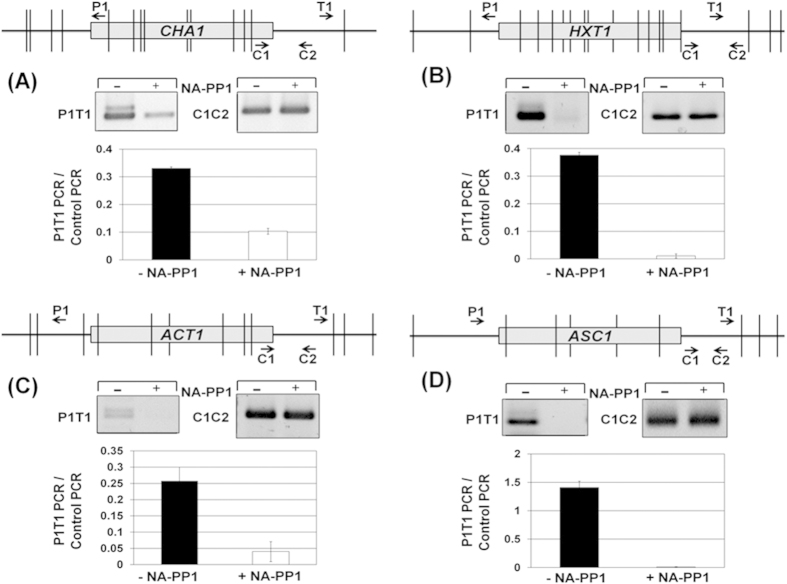
Gene looping is compromised in the absence of Kin28 kinase activity. Top panels in (**A–D**) represent a scaled schematic depiction of genes indicating the positions of AluI and NlaIV restriction sites (vertical lines) and the PCR primers (arrows) used for CCC analysis. (A – D), CCC analysis of *CHA1*, *HXT1*, *ACT1* and *ASC1* in kin28-as mutant in the absence of NA-PP1 (-NA-PP1) or in the presence of NA-PP1 (+NA-PP1). P1T1 PCR reflects the looping signal and the C1C2 PCR reflects the loading control indicating that an equal amount of template DNA was used in each CCC PCR reaction. The gel figures shown here are cropped to indicate the bands specific for P1T1 and C1C2 PCR signals used in quantification.

We have previously demonstrated simultaneous crosslinking of general transcription factors and termination factors to both the 5′ and 3′ ends of a transcriptionally active gene^18^,^21^,^34^,^41^. We also showed that the simultaneous terminal end occupancy of initiation and termination factors is accompanied by a physical interaction of the promoter and terminator regions of a gene resulting in the formation of looped gene architecture^18^,^21^,^34^,^41^. We found a similar crosslinking of Rna15, Ssu72 and Kin28 to both the ends of all four genes tested in this analysis in the absence of NA-PP1 (Fig. 2). In the presence of NA-PP1, however, in addition to loss of 3′ end occupancy, 5′ end occupancy of both Rna15 and Ssu72 registered a 30-90% decline. The crosslinking of Kin28 to both the ends of a gene similarly decreased in the absence of its kinase activity. These results strongly suggested that all six genes examined here assumed a looped conformation in a manner dependent on Kin28 kinase activity. We therefore carried out CCC analysis of *CHA1*, *HXT1*, *ACT1* and *ASC1* in Kin28-as cells in the presence and absence of NA-PP1. We have previously used this approach to demonstrate looping of genes in a transcription-dependent manner^18^. In CCC assay, the physical interaction of the promoter and terminator regions of a gene is converted into a PCR product obtained using primers flanking the promoter (P1 primer) and the terminator (T1) regions as shown in Fig. 5 (top panel)^42^. CCC analysis revealed that *CHA1*, *HXT1*, *ACT1* and *ASC1* assume a looped gene conformation during transcription in the Kin28-as mutant as indicated by a strong P1T1 PCR signal for all four genes (Fig. 5A-D, -NA-PP1 lane and black bars). In the presence of NA-PP1, however, the looped gene architecture of all four genes was abrogated as there was a 3–40 fold decline in P1T1 PCR signal for different genes (Fig. 5A-D, +NA-PP1 lane and white bars). A logical conclusion of these results is that the kinase activity of Kin28 is essential for gene looping.

### Kin28 kinase-dependent gene looping facilitates termination of transcription

The results described above clearly demonstrate that both termination of transcription and gene looping are dependent on the Kin28 kinase activity. The observation raises the possibility of Kin28 kinase bringing about termination of transcription through gene looping. If gene looping is indeed facilitating termination of transcription, then disruption of gene looping by mutating a factor other than Kin28 will also lead to a termination defect. We therefore used the looping defective TFIIB mutant *sua7-1* to check our hypothesis. In the *sua7-1* mutant, the recruitment of TFIIB at the promoter and the initiation of transcription remain unaffected. The 3′ end occupancy of TFIIB, however, is severely compromised in the mutant^26^. Consequently, the *sua7-1* mutant is defective in gene looping^26^. We have previously used this strain to demonstrate that Srb5-dependent gene looping facilitates termination of transcription of a subset of genes^23^. To determine if Kin28 kinase-mediated gene looping is similarly affecting termination of transcription, we performed strand-specific TRO analysis of *CHA1*, *HXT1*, *ACT1* and *ASC1* in the looping defective *sua7-1* strain. Our results show that the polymerase reads through the terminator region of all four genes in the looping defective strain (Supplemental Figs 7B, 7C, 7D and 7E, white bars). No such read-through was observed in the isogenic wild type strain (Supplemental Figs 7B, 7C, 7D and 7E, black bars). This termination defect occurred even in the presence of enzymatically active Kin28. Furthermore, crosslinking of Kin28 towards the 3′ end of genes was severely compromised in *sua7-1* cells (Supplemental Fig. S8). On the basis of these results, we conclude that it is not Kin28 kinase activity per se, but the kinase-mediated gene looping that facilitates termination of transcription.

## Discussion

Since its discovery, the function of CTD kinase activity of TFIIH in transcription has been the focus of intense scrutiny. Using the analog-sensitive mutant of Kin28, it has been demonstrated that Kin28 kinase is neither essential for transcription nor for survival of yeast cells^12^,^14^. Our results suggest that Kin28 kinase is not an absolute requirement for transcription, but is required for optimal transcription of genes. The prevailing view is that the general transcription factors are required for the transcription of a vast majority of RNAPII-dependent genes^43^,^44^,^45^. This may not be entirely true. A recent study revealed that the TFIIB is required for transcription of only a subset of genes in humans^46^. It is possible that the Kin28 kinase activity of TFIIH is also not necessary for transcription of all RNAPII-transcribed genes. If the TFIIH-kinase is required for transcription of a subset of non-essential genes, the cell may still be viable in the absence of the kinase activity, but the cell fitness may be adversely affected. This may explain why a defect in Kin28 kinase does not affect the cell viability and global poly(A)-mRNA level drastically, but is still necessary for normal growth of yeast cells. Alternatively, Kin28 kinase may be affecting transcription of protein coding genes on a genomewide scale, but compensatory mechanisms such as a parallel decrease in the mRNA degradation rate is buffering the global transcript level^47^,^48^,^49^,^50^.

The Kin28 kinase occupies both the 5′ and the 3′ ends of a number of genes^51^,^52^. Most studies have focused on the role of TFIIH kinase at the 5′ end of genes. Here we demonstrate a novel role of Kin28 kinase in termination of transcription. We provide several lines of evidence in support of our claim. First, RNAPII reads through the termination signal into the downstream region under the kinase-defective condition (Fig. 1). Second, recruitment of CF1 and CPF termination complexes towards the terminator end of gene is compromised in the absence of kinase activity (Fig. 2). Third, localization of Kin28 at the 3′ end of genes is dependent on the kinase activity of protein (Fig. 3). Genomewide RNAPII ChIP analysis has been performed in the presence and absence of Kin28 kinase activity in budding yeast^5^,^53^. These studies revealed the role of Kin28 kinase in the promoter escape step of transcription of a subset of genes. To corroborate the role Kin28 kinase in termination of transcription, we compared our results with the genomewide data published in these two studies. We extracted the RNAPII-ChIP data for four genes used in our study; *CHA1, HXT1, ACT1* and *ASC1,* from Struhl’s published genomewide database (Supplemental Fig. S9). All four genes exhibit a decrease in polymerase density in the coding region in the absence of Kin28 (Supplemental Fig. S9). More importantly, there was about 1.5 to 2.5 fold increase in the polymerase density beyond the 3′ end of all four genes in the absence of Kin28 (Supplemental Fig. S9). These results are in broad agreement with the analysis presented in this study. Even *CHA1*, which is expressed at a very low level under the condition Struhl’s ChIP-Seq analysis was performed, exhibited an increase in polymerase signal towards the 3′ end of the gene in the absence of Kin28 (Supplemental Fig. S9 A). We, however, could not find a correlation between Kin28-dependent termination and the SAGA-dependence of genes. In Bentley’s study, we found a slightly higher RNAPII signal near the 3′ end of long genes in the absence of Kin28 kinase activity^53^. Whether the higher polymerase density beyond the 3′ end of long genes in the absence of Kin28 kinase reflects a termination defect, however, needs further investigation. Inhibition of Cdk7, which is the human homologue of Kin28, similarly affects termination of transcription of several genes^54^. Thus, the role of TFIIH kinase in termination could be an evolutionarily conserved feature of transcription by RNAPII in eukaryotes.

TFIIH is not the only general transcription factor that has been implicated in termination of transcription. A similar termination function has been found for TFIIB as well. Just like Kin28, TFIIB cross-links to the 3′ end of genes and facilitates recruitment of the termination factors there^26^. Although, a role of TFIIB in termination has yet to be established in budding yeast, it has already been demonstrated in mammals and flies^55^,^56^. We have recently demonstrated the role of another initiation factor, Mediator complex, in the termination of transcription^23^,^41^. The emerging view is that the initiation and termination factors do not have exclusive roles in the initiation and termination steps of transcription respectively. We have shown that at least some initiation factors participate in the termination of transcription, while additionally, some termination factors function in the initiation/reinitiation of transcription^34^.

We have previously demonstrated that gene looping facilitates interaction of the promoter-bound factors with the 3′ end of genes, and of terminator-bound factors with the 5′ end of genes^18^,^21^,^23^,^34^. We hypothesize that it is gene looping that allows a transcription factor to function at both the ends of a gene. The termination function of TFIIB in flies and mammalian system is completely dependent on its interaction with the 3′ end of a gene^55^,^56^. Here we show that Kin28 crosslinking to the 3′ end of a gene, and its consequent role in termination of transcription may also be dependent on gene looping. Previously, we have shown that Srb5-dependent gene looping helps in termination of transcription^41^. We have also shown that gene looping is lost in the mutants of termination factors that are defective in termination^21^,^34^. On the basis of these results we propose that gene looping is playing a general role in termination of transcription in budding yeast. Accordingly, we show that TFIIB-dependent gene looping similarly affects the termination of transcription of the same set of genes that are affected by Kin28 kinase. Although, gene looping has been shown for only a few genes in higher eukaryotes, TFIIB similarly crosslinks to the 3′ end of genes in mammalian cells as well and facilitates termination of transcription^56^. The crosslinking of TFIIB to both the ends of genes in mammalian systems strongly suggest that genes are in looped conformation. The possibility of gene looping playing a similar role in termination of transcription in higher eukaryotes cannot be ruled out.

## Methods

### Yeast Strains

The yeast strains used in this study are listed in supplemental Table S1. Strains BPM5, SAM89 and SAM90, which contain C-terminal TAP-tagged TFIIH subunits Tfb4, Kin28 and Ssl2 respectively, were constructed by transforming the wild type strain BY4733 with DNA that was PCR amplified from plasmid pBS1539 (*URA* marker). Strains SAM99, SAM107 and ZD3, which contain C-terminal TAP-tagged TFIIH subunits Kin28, Ssl2, and Tfb4 respectively, were constructed by transforming the Kin28as mutant yFR763 with DNA that was PCR amplified from plasmid pBS1479 (*TRP* marker). The C-terminal Myc-tagged strains SAM93, SAM51, SAM94, SAM103, SAM101, SAM102, SAM104 and SAM105 which contain Myc tagged Rna15 or Ssu72, respectively, were created by transforming either wild type or Kin28as cells with DNA that was PCR amplified from plasmid pFA6-13Myc-HIS3mx6. Strain SAM106, which contains a C-terminal Myc tagged Rpb4 was created by transforming Kin28as cells with DNA that was PCR amplified from plasmid pFA6-13Myc-HIS3mx6. Strain AS1 was derived from BY4733 as described in^57^.

### Cell Culture and Kinase Inhibition with NA-PP1

Kin28-as mutant cells were grown in uracil-depleted media to an *A*_600_ 0.6 as previously described^21^. Cell cultures were then divided evenly and transferred to 100 ml of fresh media that either contained 25 μl of DMSO (control) or DMSO + NAPP1 (Final Concentration 7 μM NA-PP1). The induction and inhibition of kinase activity occurred simultaneously for the inducible genes. The cells were grown under inducing and +/– NAPP1 conditions for either 30, 60 or 90 minutes during the time course experiments. In all other experiments, the cells were grown under inducing +/– NAPP1 conditions for one hour. In figures that depict a time 0 measurement, an equal aliquot of cells was removed prior to induction and kinase inhibition. For constitutively expressed genes, cells were grown to *A*_600_ 0.6 and then grown in the presence and absence of NA-PP1 as described above.

### Capture Chromosome Conformation Assay (CCC)

CCC experiments were performed as described previously^42^.

### Chromatin Immunoprecipitation (ChIP)

ChIP experiments were performed as described previously^21^.

### Transcription Analysis

Transcription analysis was performed by the RT-PCR approach as described previously^18^.

### Strand-Specific ‘Transcription Run On’ (TRO) Assay

The strand specific ‘Transcription Run-On’ (TRO) assay was performed by the modification of method described in Core *et al.*^58^. The protocol can be divided into three steps:

### Step I. Labeling and isolation of nascent RNA with Brd-UTP

Cells were grown as described above. The cell pellet was washed with 10 ml of ice cold TMN buffer (10 mM Tris-HCl pH 7.5, 5 mM MgCl_2_, 100 mM NaCl) and resuspended in 940 μl of DEPC (Diethylpyrocarbonate)-treated ice cold water. Cells were permeabilized by the incubating with 60 μl 10% sarkosyl at 4 °C for 25 minutes with gentle shaking. Permeabilized ells were spun down by centrifugation at 1.2×g for 6 minutes at 4 °C. The cells were resuspended in 150 μl of transcription elongation reaction buffer (50 mM Tris-HCl pH 7.5, 100 mM KCl, 10 mM MgCl_2_, 2 mM DTT, 0.75 mM each of ATP, CTP, GTP and Brd-UTP, 5 μl of RNAse inhibitor cocktail from NEB) and incubated at 30 °C for 5 minutes. Cells were immediately lysed by addition of 500 μl ice cold Trizol and 250 μl acid-washed glass beads followed by vigorous shaking at 4 °C for 20 minutes. The lysate was transferred to a new 1.5 ml microfuge tube containing 500 μl of Trizol and 200 μl of chloroform. The tubes were shaken vigorously and centrifuged at 14000 rpm at 25 °C for 20 minutes. The supernatant was extracted with phenol-chloroform three times. Total RNA was precipitated by incubating with NaCl overnight at −20 °C. The RNA pellet was washed once with ice cold 70% ethanol and resuspended in 100 μl of DEPC-treated water. RNA was further purified using the QIAGEN RNA Easy kit and eluted twice with 50 μl of DEPC treated water.

### Step II. Purification of Brd-UTP-labeled RNA

About 25 μl of anti-BrdU-conjugated agarose beads were washed three times with 500 μl of binding buffer (0.25× SSPE buffer, 1 mM EDTA, 0.05% Tween20, 37.5 mM NaCl). Beads were blocked using 500 μl of blocking buffer (485 μl binding buffer containing 5 μl of 10% polyvinylpyrolidone, 10 μl of Ultrapure BSA, Sigma) by gently shaking for 1 hour at 4 °C. Beads were washed two times with 500 μl of binding buffer and then 400 μl of binding buffer was added to the beads and placed on ice.

The RNA from step I was incubated at 65 °C water bath for 5 minutes and immediately placed on ice for 2 minutes. The RNA was then incubated with the processed anti-BrdU beads with gentle shaking for 2 hours at 4 °C. Beads were then washed sequentially with 500 μl of binding buffer, 500 μl of low salt buffer (0.2× SSPE, 1 mM EDTA, 0.05% Tween20) 500 μl of high salt buffer (0.25× SSPE, 1 mM EDTA, 0.05% Tween-20, 100 mM NaCl) and 500 μl of TET buffer (1×TE buffer, 0.05% Tween20). RNA was eluted two times with 150 μl of elution buffer (20 mM DTT, 150 mM NaCl, 50 mM Tris-HCl pH 7.5, 1 mM EDTA, 0.1% SDS) by incubating at 42 °C for 5 minutes followed by a final elution with 200 μl of elution buffer at 42 °C for 5 minutes. After phenol-chloroform extraction, RNA was precipitated overnight at -20 °C in the presence of NaCl. RNA pellet was resuspended in 26 μl of DEPC-treated water and quantified using a Nanodrop spectrophotometer. RNA concentration was adjusted to 50 ng/μl.

### Step III. Reverse transcription and PCR amplification of RNA

The cDNA synthesis was performed with Protoscript II (NEB) using the strand specific primers following to manufacturer’s recommendation. PCR amplification was performed using either Advantage polymerase or Taq polymerase depending on amplicon size. Quantification was done as described in Medler *et al.*, (2011).

### Coimmunoprecipitation (CO-IP)

CO-IP was performed as described previously^21^.

### Western Blot Analysis

Western blotting was performed as described previously^21^. Anti-CBP and anti-Myc antibodies were from Upstate Biotechnology. Anti-CTD antibodies 3e10, 3e8 and 4e12 that detect CTD-serine-2-P, CTD-serine-5-P and CTD-serine-7-P respectively were purchased from Millipore.

### Data Analysis

The data shown in figures is the result of at least four biological replicates. The data was quantified and statistical analysis was performed as described in^42^. Error bars represent one unit of standard deviation.

## Additional Information

**How to cite this article**: Medler, S. and Ansari, A. Gene looping facilitates TFIIH kinase-mediated termination of transcription. *Sci. Rep.* **5**, 12586; doi: 10.1038/srep12586 (2015).

## Supplementary Material

Supplementary Information
