# Supplementary material for: Gene looping facilitates TFIIH kinase-mediated termination of transcription
Source: Sci Rep. 2015 Aug 19;5:12586. doi: 10.1038/srep12586 (PMC4541409; doi:10.1038/srep12586)
Supplement: Supplementary Information [file srep12586-s1.pdf]

Gene looping facilitates TFIIH kinase-mediated termination of transcription

Scott Medler[1] and Athar Ansari[1*]

[1]Department of Biological Science, Wayne State University, 5047 Gullen Mall
Detroit, MI 48202

[*]To whom correspondence should be addressed. Phone: (313) 577-9251; Fax: (313) 571-6891;
E-MAIL: ansari@biology.biosci.wayne.edu

Table S1: Yeast Strains

BY4733	MATa his3Δ200 trp1Δ63 leu2Δ0 met15Δ0 ura3Δ0	Hampsey lab
SAM89	MATa his3Δ200 trp1Δ63 leu2Δ0 met15Δ0 ura3Δ0, KIN28-TAP, URA+	This study
SAM93	MATa his3Δ200 trp1Δ63 leu2Δ0 met15Δ0 ura3Δ0, KIN28-TAP, URA+, RNA15-13xMyc, TRP+	This study
SAM51	MATa his3Δ200 trp1Δ63 leu2Δ0 met15Δ0 ura3Δ0, RNA15-13xMyc, TRP+	El Kaderi et al, 2009(1)
SAM94	MATa his3Δ200 trp1Δ63 leu2Δ0 met15Δ0 ura3Δ0, KIN28-TAP, URA+, SSU72-13xMyc, TRP+	This study
SAM103	MATa his3Δ200 trp1Δ63 leu2Δ0 met15Δ0 ura3Δ0, SSU72-13xMyc, TRP+	This study
yFR763	MATα, ade::higG, his3Δ200, leu2Δ0, lys2Δ0,met15Δ0,trp1Δ63,ura3Δ0 kin28::kin28-L83G [pSH579, ARS CEN URA3 kin28-L83G]	Liu Y et al, 2004(2)
SAM99	MATα, ade::higG, his3Δ200, leu2Δ0, lys2Δ0,met15Δ0,trp1Δ63,ura3Δ0 kin28::kin28-L83G [pSH579, ARS CEN URA3 kin28-L83G] Kin28as-TAP, TRP+	This study
SAM101	MATα, ade::higG, his3Δ200, leu2Δ0, lys2Δ0,met15Δ0,trp1Δ63,ura3Δ0 kin28::kin28-L83G [pSH579, ARS CEN URA3 kin28-L83G] Kin28as-TAP, TRP+, RNA15-13xMyc, HIS+	This study
SAM102	MATα, ade::higG, his3Δ200, leu2Δ0, lys2Δ0,met15Δ0,trp1Δ63,ura3Δ0 kin28::kin28-L83G [pSH579, ARS CEN URA3 kin28-L83G] Kin28as-TAP, TRP+, SSU72-13xMyc, HIS+	This study
SAM104	MATα, ade::higG, his3Δ200, leu2Δ0, lys2Δ0,met15Δ0,trp1Δ63,ura3Δ0 kin28::kin28-L83G [pSH579, ARS CEN URA3 kin28-L83G] RNA15-13xMyc, HIS+	This study
SAM105	MATα, ade::higG, his3Δ200, leu2Δ0, lys2Δ0,met15Δ0,trp1Δ63,ura3Δ0 kin28::kin28-L83G [pSH579, ARS CEN URA3 kin28-L83G] SSU72-13xMyc, HIS+	This study
SAM106	MATα, ade::higG, his3Δ200, leu2Δ0, lys2Δ0,met15Δ0,trp1Δ63,ura3Δ0	This study

	kin28::kin28-L83G [pSH579, ARS CEN URA3 kin28-L83G] RPB4-13xMyc, HIS+	
AS1	MATa, his3Δ200, trp1Δ63, leu2Δ0, met15Δ0, ura3Δ0, *sua7-1*	This study

References:

1. **El Kaderi, B., Medler, S., Raghunayakula, S., and Ansari, A. (2009) *J Biol Chem* 284, 25015-25025**
2. **Liu, Y., Kung, C., Fishburn, J., Ansari, A. Z., Shokat, K. M., and Hahn, S. (2004) *Mol Cell Biol* 24, 1721-1735**

Table S2: Primer Tables

cDNA

Name	Sequence
Oligo dT	TTTTTTTTTTTTTTTTTTTTTTTTT
18s	GACGGAGTTTCACAAGATTACC
CHA1 d1	GGAAAAAATCAATACTAGCAAAATA
CHA1 d2	CCCCTTTATACAAATTCTGTGC
CHA1 d3	GCTTTTCTTCACTTAGTAAGGATTAA
CHA1 d4	CTGGGGTCTTCATTTGTGTCA
CHA1 d5	GTGCTGGAAATGGCAAACG
CHA1 d6	CAGCTGAGTAACTAACTCTCATGG
CHA1 d7	TAGTTTTTAGTACGATTGCATTGTC
ACT1 d1	ATACGCGCACAAAAGCAGAG
ACT1 d2	AATTTTCGTAGAAAAGGGAGAGAC
ACT1 d3	GGTATCAAAACGCCGGACTC
ACT1 d4	TTTGCGTAACGTTTGGATGG
ACT1 d5	CGCATTATTTGTGAAGTATGGTC
ACT1 d6	AGTGAAGCTCAGGAAATTCAGC
ACT1 d7	AACTCCTTTACAACAAGGTTTACTC
HXT1 d1	TTGTTTTAACACCAATATAATATCACC
HXT1 d2	GTAACACTACTAGATGTTGAAGCAGC
HXT1 d3	GGTAGCGTGCATTTTCTCGTC
HXT1 d4	GAACATTTACATGAAGAGGGCTG
HXT1 d5	CAAATTGATGCGGTAGCGTAG
HXT1 d6	GGTTTGGGCATTGTTTTTCC

HXT1 d7	GATTCTTCGTGATTCTCGTCG
ASC1 d1	GAACTTTATACATATTCTTAGTTAGCAGTC
ASC1 d2	TGTACATATGTATTTTCGCAGCA
ASC1 d3	GCCAAGGAGACTGAATTTAATG
ASC1 d4	CTATGGAATGGGGGTTTTAAG
ASC1 d5	GGTTATGGCAGACATGCCAC
ASC1 d6	CAAAGTTGATGAGAGTGAAAGAGG
ASC1 d7	CTCGACCACAGATGGCACC

18s Control-PCR

Name	Sequence
18s F	GGAATAATAGAATAGGACGTTTGG
18s R	GTTAAGGTCTCGTTCGTTATCG

Gene Specific RT-PCR Primers

Name	Sequence
CHA1 F	AATTCAAAAGGACGGTAAAAGAT
CHA1 R	AAGGGATGAACATAAATGGGC
CHA1-TO	CAGTCGTGGTTCCTACAGCG
MET16 F	CATTTGGTTTGACTGGCTTGG
MET16 R	TCGTACTTGTCATCATCTTTCTCC
INO1 F	GATATCCAGAATTTCAAAGAAGAAAAC
INO1 R	TATTCTGCGGTGAACCATTAATATAG
ACT1 F	TACTCTTTCTCCACCACTGCTG
ACT1 R	GATTTCCTTTTGCATTCTTTCG
ACT1-TO	GAAATATTTTCGTGATAAGTGATAGTG
MSN5 *F*	CAATGCCAATCCAAACAGTG

MSN5 R	CGCACTATTACACAGCACATTTA
CMP1 F	AACCGCAGAATAATGAATAAAGTG
CMP1 R	GATATAAGGTTGGGTTCTTTGCT
SPC1 F	GTGCTCTCGCTACTTTTCTGG
SPC1 R	CATTGTGCTGTTCAGAGAACCA
IMD4 F	ATTGGTATGGGTTCTGGGTC
IMD4 R	GCCTTCAATCTCTTACCATCC
ASC1 F	CTTACGCTTTGTCTGCTTCTTG
ASC1 R	GATGGTCTTGTCACGGGAAC
ASC1-TO	AGATTCGTCGGTCACAAGTCC
GAL10 F	GATCTTCCATACAAAGTTACGGG
GAL10 R	CACAAATCTTGCGTCATAACG
HXT1 F	ATTTGGTATGAAGCACCACGA
HXT1 R	GGGCGACCTCAGATATTAGCA
HXT1-TO	ATTTGGTATGAAGCACCACGA

***HXT1* ChIP**

Name	Sequence
HXT1 P	GCGATGAGATAAGATAAAAGGGA
	GATTACCGATTCCTCTACTTTTGA
HXT1 M	ATTTGGTATGAAGCACCACGA
	GGGCGACCTCAGATATTAGCA
HXT1 T	GGTGGAAACGAATGGATGTC
	TCTTAGTGTTGTAACCCAAATGC

***CHA1* ChIP**

Name	Sequence
CHA1 P	GCCCCAGCGGAAATGTAA
	CATTCATATTTCAAGAAAAATTGTG
CHA1 M	GCCCAGGTTATCGTGAGTG
	CACCTCCACCAACGCTGC
CHA1 T	GGAAGAAGCGTTGGATAGCAT
	CAGTAGTTTATGCTTTATGCTCG
CHA1 D1	GCACAGAATTTGTATAAAGGGG
	GCTTTTCTTCACTTAGTAAGGATTAA
CHA1 D2	GTTCCGTAATAATCTTCCCAGC
	CTGGGGTCTTCATTTGTGTCA

***ASC1* ChIP**

Name	Sequence
ASC1 P	GACTGCTCCTTTGGTTTTCC
	GGTTGACCAGCAGAAGTAGCC
ASC1 M	CGAAAAAGCTGATGATGACTCTG
	TTGATGTTGGAGTTGTGACCG
ASC1 T	TGGCAAGTTATGACTGCTAACTAAG
	GCCAAGGAGACTGAATTTAATG

***ACT1* ChIP**

Name	Sequence
ACT1 P	CAAACTCGCCTCTCTCTCTCC
	GCAAGCGCTAGAACATACCAG
ACT1 M	CATACCTTCTACAACGAATTGAGAG
	CTTCATCAAGTAGTCAGTCAAATCTC

ACT1 T	TGGTCCATCTATCGTTCACCA
	ATAAAACTGAAAAGCGATGAAGAG
ACT1 D1	GTTTTGTCTCTCCCTTTTCTACG
	GGTATCAAAACGCCGGACTC
ACT1 D2	CCGCCATTAGAATTTGAGTCC
	TTTGCGTAACGTTTGGATGG

HXT1 CCC

Name	Sequence
HXT1 P	TCGGGTGTTAAGAAATATTTTGC
HXT1 T	TTAACAGATAACCGAGTCGATCTC
HXT1 C1	AATACCACATAGGCGCTATACATAG
HXT1 C2	CGTCTTTTCTTTACTGCTTCACC

CHA1 CCC

Name	Sequence
CHA1 P	GGAAAATGTTTATACAGTTTTCTCTT
CHA1 T	GGAAAATGTTTATACAGTTTTCTCTT
CHA1 C1	GGAAGAAGCGTTGGATAGCAT
CHA1 C2	CGTTTTGGATATGTTGATGCTTAC

ACT1 CCC

Name	Sequence
ACT1 P	CGAGTTTGGTTTCAAAACGG
ACT1 T	CCGCCATTAGAATTTGAGTCC
ACT1 C1	TGGTCCATCTATCGTTCACCA
ACT1 C2	AATTTTCGTAGAAAAGGGAGAGAC

ASC1 CCC

Name	Sequence
ASC1 P	GACTGCTCCTTTGGTTTTCC
ASC1 T	CTTTATTTCCTTTATTGTGGTATTAG
ASC1 C1	ATGCTGTTTCTTTGGCTTGG
ASC1 C2	TGTACATATGTATTTTCGCAGCA

C-Terminal Tagging Primers

Name	Sequence
F2- Myc-RNA15	CTATTTGGGACTTAAAACAAAAAGCATTAAGGGGAGAA TTTGGTGCATTTCGGATCCCCGGGTTAATTAA
R1- Myc-RNA15	ATCATTGCGGAACCGCATTTTTTTTTTGTATTTTTGCCTCC CTAGTTTCAGAATTCGAGCTCGTTTAAAC
RNA15-Myc-Diag	TCCAGGCCGCAAGAAGAG
F2-HA-TFIIB	TTGCTAATGGTGTAGTGTCTTTGGATAACTTACCGGGCG TTGAAAAGAAACGGATCCCCGGGTTAATTAA
R1-HA-TFIIB	CACGAGTACCCGTGCTTCTTGTTCCTATAATTTACTGTTT TATCACTTCAGAATTCGAGCTCGTTTAAAC
TFIIB-HA-Diag	CCGATGCAAGTCACTACTTCTG
Myc-tag-Diag	CAAGTCTTCCTCGGAGATTAGC
HA-tag-Diag	GGTAGAGGTGTGGTCAATAAGAGC
F2 KIN28-Myc/HA-	TCAAAGAATTACCACCACCAAGTGACCCGTCTTCAATAAAAATACGTAACCGG ATCCCCGGGTTAATTAA
R1 KIN28-Myc/HA-	GATACATCTAATGTCAATAACACAGATTCTACAAATTTTATAAAATCATAGAATT CGAGCTCGTTTAAAC
5’ KIN28-Tag-Diag	CGCCTTAGATTTTATGTGTGGA
F2-SSU72-	GGCAAAGCTCACATTCTCAACTACCGTCATTATACGCTCCTTCATATTACCGG ATCCCCGGGTTAATTAA

Myc-	
R1-SSU72-Myc-	ATGAGGGCCGCTTAATGCTTATGCTTTTCTACAGTAATTGACCGTTTTGTGAATTCGAGCTCGTTTAAAC
5' SSU72 –Tag Diag	ATGATGATGAAAATGCTAAAATTG
5' TFB4-C-TAP	GGAAACCAGTTGTTCCAAGGTTGAAAGCCAAAAAGAAGGTGACGAAACCATCCATGGAAAAGAGAAG
3' TFB4-C-TAP	ATTGTGACGAAGGTTACCTGCTTGAAACGGATAATGTTCATTCCTTTCGTTACGACTCACTATAGGG
TFB4-TAP-DIAG	AGGACATCATGCTATTTAACAGGG
5' C-TAP Ssl2	AGGAACATCATCCATTAATCAGAAAGATGTATTATAAGAATTTGAAGAAGTCCATGGAAAAGAGAAG
3'C-TAP Ssl2	TATGACTGAATAGATTCAAAATAGGAAGGTGACAATGAAACCAAGCCTATTACGACTCACTATAGGG
5'C-TAP SSL2-Diag	TTACACATTTACACGGAATGGAG
5'C-TAP kin28	TCAAAGAATTACCACCACCAAGTGACCCGTCTTCAATAAAAATACGTAACTCCATGGAAAAGAGAAG
3'C-TAP kin28	GATACATCTAATGTCAATAACACAGATTCTACAAATTTTATAAAATCATATACGACTCACTATAGGG
5'C-TAP kin28-Diag	CGAAGTTTCTTCCTTTATGACG
5'RNA15-TAP-C	CTATTTGGGACTTAAAACAAAAAGCATTAAGGGGAGAATTTGGTGCATTTTCCATGGAAAAGAGAAG
3'RNA15 –TAP-C	ATCATTGCGGAACCGCATTTTTTTTTTGTATTTTTGCCTCCCTAGTTTCATACGACTCACTATAGGG
5'TAP-Rna15-D	TCCAGGCCGCAAGAAGAG
5'Ssu72-C-TAP	GGCAAAGCTCACATTCTCAACTACCGTCATTATACGCTCCTTCATATTACTCCATGGAAAAGAGAAG
3'Ssu72-C-TAP	ATGAGGGCCGCTTAATGCTTATGCTTTTCTACAGTAATTGACCGTTTTGTTACGACTCACTATAGGG
5'Ssu72-C-TAP-Diag	GTGAAGATTTGATGAATAGAGG
3' TAP Diag	GTTGAATTTGTTGTCTACTTTCGG

Supplemental Fig. S1

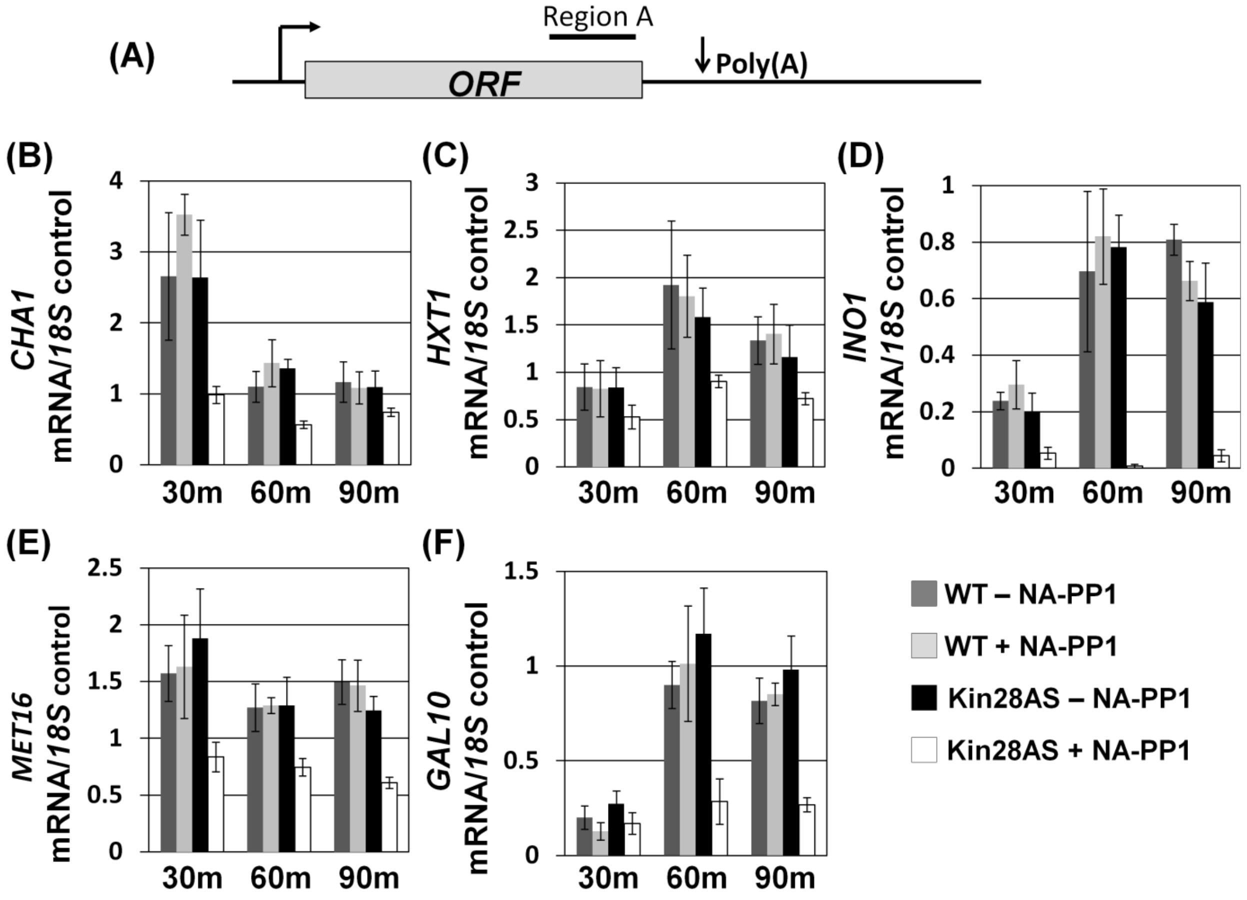

Figure S1. The steady state mRNA levels of inducible genes are reduced in the absence of Kin28 kinase activity. *A*, Schematic depiction of a gene indicating the region of cDNA amplified in the RT-PCR assay. *B-F,* Quantification of mRNA levels of *CHA1*, *HXT1*, *INO1*, *MET16* and *GAL10* in the wild type and analog-sensitive Kin28 mutant (kin28-as) following induction of transcription. Upon induction, cells were grown with either DMSO (-NA-PP1) or 7 µM NA-PP1 (+NA-PP1) for the indicated period of time. The transcript level of 18S was used as the control for the normalization of results. Error bars represent one unit of standard deviation. The results shown here are averages of at least four PCRs from two independent RNA preparations.

Supplemental Fig. S2

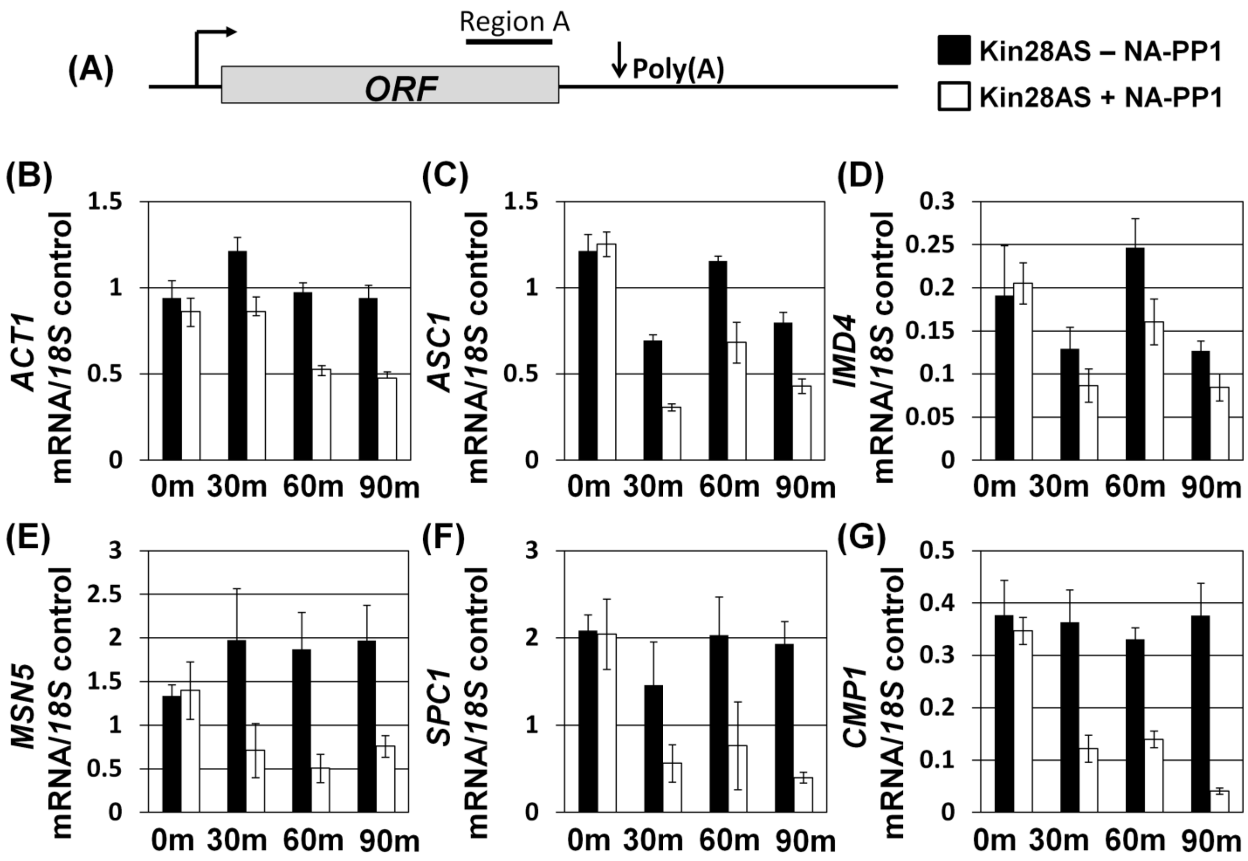

Figure S2. The steady state mRNA levels of non-inducible genes are reduced in the absence of Kin28 kinase activity. *A*, Schematic depiction of a gene indicating the region of cDNA amplified in the RT-PCR assay. *B-G,* Quantification of mRNA levels of *ACT1*, *ASC1*, *IMD4*, *MSN5*, *SPC1* and *CMP1* genes in the analog-sensitive Kin28 mutant (kin28-as) in the absence of (-NA-PP1) or presence of NA-PP1 (+NA-PP1). The transcript level of 18S was used as the control for normalization of results. Error bars represent one unit of standard deviation. The results shown here are averages of at least four PCRs from two independent RNA preparations.

Supplemental Fig. S3

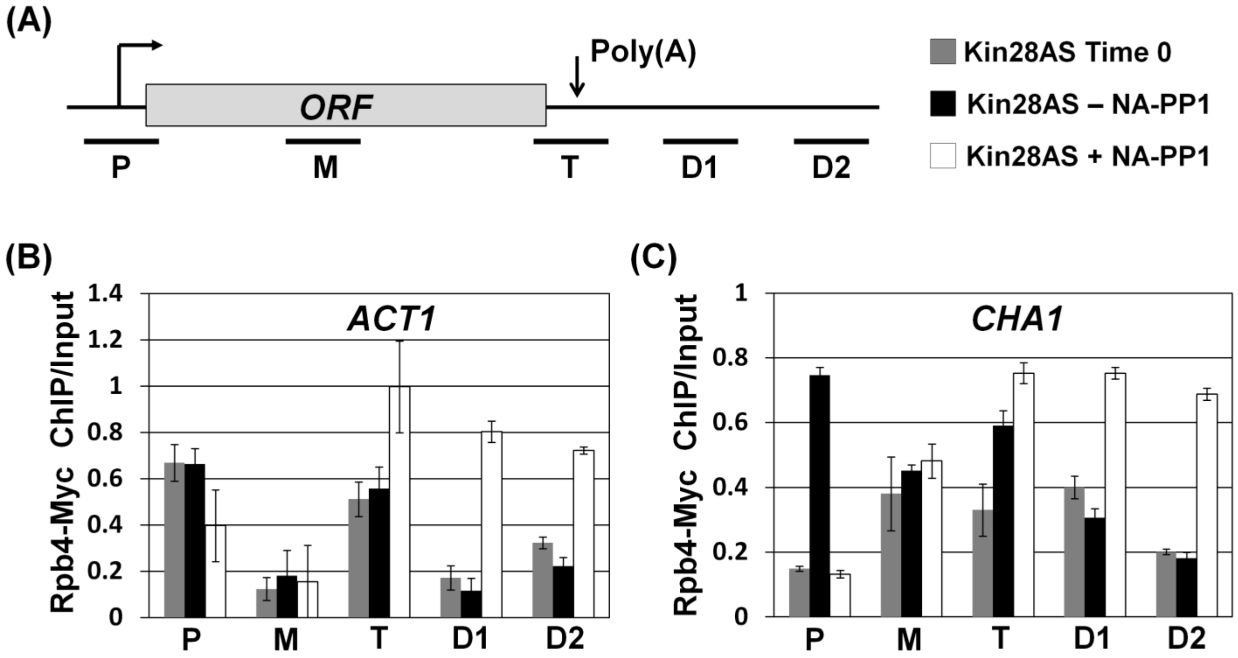

Figure S3. RNAPII density measured in terms of Rpb4-ChIP showing the readthrough of polymerase beyond the poly(A) site in the absence of Kin28 kinase activity. *A*, Schematic depiction of a gene indicating different regions P, M, T, D1 and D2 of a gene PCR amplified in the ChIP assay. *B-C,* Quantification of Rpb4-ChIP signals in the analog-sensitive Kin28 mutant (kin28-as) in the absence (-NA-PP1) or presence (+NA-PP1) of NA-PP1. Input represents DNA prior to immunoprecipitation. Error bars represent one standard deviation. The results shown here are averages of at least four PCRs from two independent ChIP experiments.

Supplemental Fig. S4

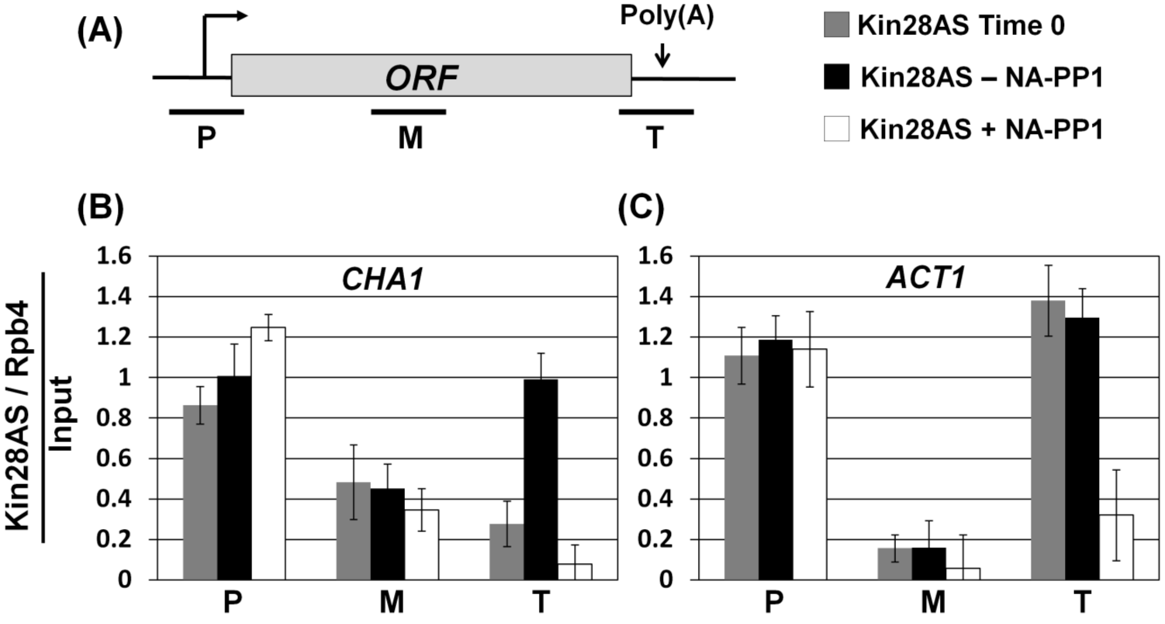

Figure S4. Inhibition of Kin28 Kinase alters the ratio of Kin28/RNAPII at the terminator regions for *CHA1* and *ACT1* genes. *A*, Schematic depiction of a gene indicating the position of the promoter (P), coding (M) and terminator (T) regions PCR amplified in the ChIP assay. *B – C,* Quantification of Kin28AS ChIP signals, shown in Fig. 3, normalized with Rpb4-Myc signals, shown in Fig. S3, in the kin28-as mutant in the absence of NA-PP1 (-NA-PP1) or in the presence of NA-PP1 (+NA-PP1). Input represents DNA prior to immunoprecipitation. Error bars represent one standard deviation.

Supplemental Fig. S5

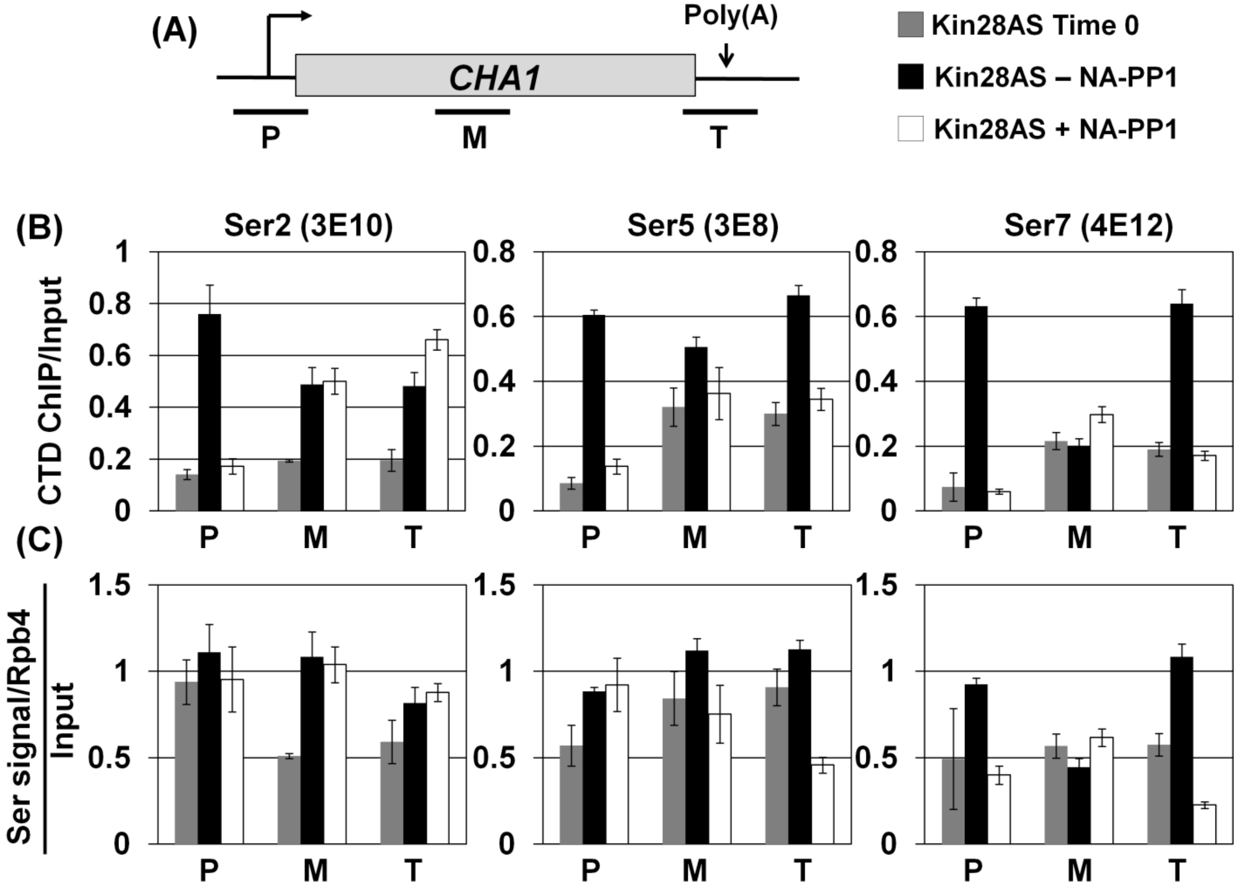

Figure S5. Inhibition of Kin28 Kinase alters CTD phosphorylation pattern along *CHA1*. *A*, Schematic depiction of a gene indicating the position of the promoter (P), coding (M) and terminator (T) regions PCR amplified in the ChIP assay. *B,* Quantification of ChIP signals for CTD-Ser2-P, CTD-Ser5-P and CTD-Ser7-P in kin28-as mutant in the absence of NA-PP1 (-NA-PP1) or in the presence of NA-PP1 (+NA-PP1). *C*, Quantification of ChIP signals for CTD-Ser2-P, CTD-Ser5-P and CTD-Ser7-P shown in panel *B* above, normalized with RNAPII signal obtained with Rpb4. Input represents DNA prior to immunoprecipitation. Error bars represent one standard deviation. The results shown here are averages of at least four PCR reactions from two independent chromatin preparations.

Supplemental Fig. S6

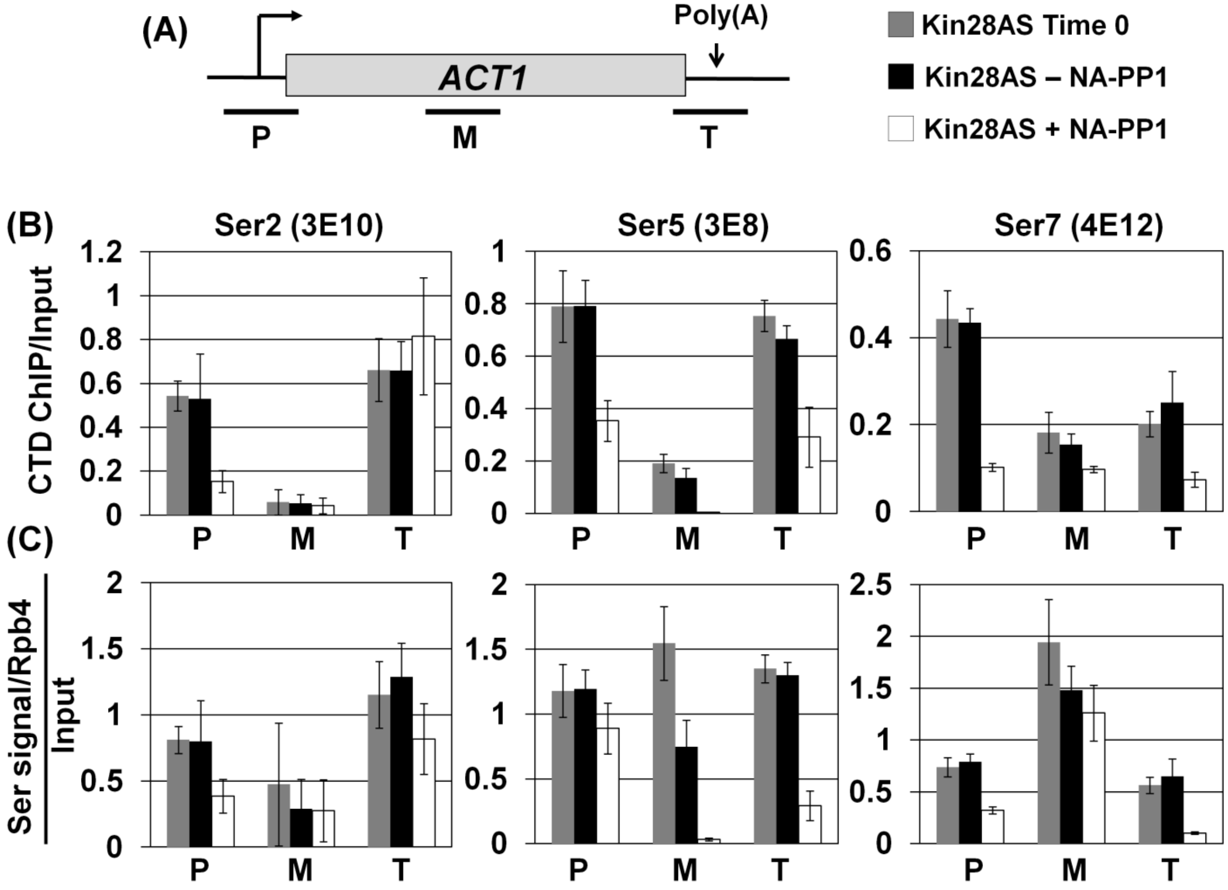

Figure S6. Inhibition of Kin28 Kinase alters CTD phosphorylation pattern along *ACT1*. *A*, Schematic depiction of a gene indicating the position of the promoter (P), coding (M) and terminator (T) regions PCR amplified in the ChIP assay. *B,* Quantification of ChIP signals for CTD-Ser2-P, CTD-Ser5-P and CTD-Ser7-P in kin28-as mutant in the absence of NA-PP1 (-NA-PP1) or in the presence of NA-PP1 (+NA-PP1). *C*, Quantification of ChIP signals for CTD-Ser2-P, CTD-Ser5-P and CTD-Ser7-P shown in panel *B* above normalized with RNAPII signal obtained with Rpb4. Input represents DNA prior to immunoprecipitation. Error bars represent one standard deviation. The results shown here are averages of at least four PCR reactions from two independent chromatin preparations.

Supplemental Fig. S7

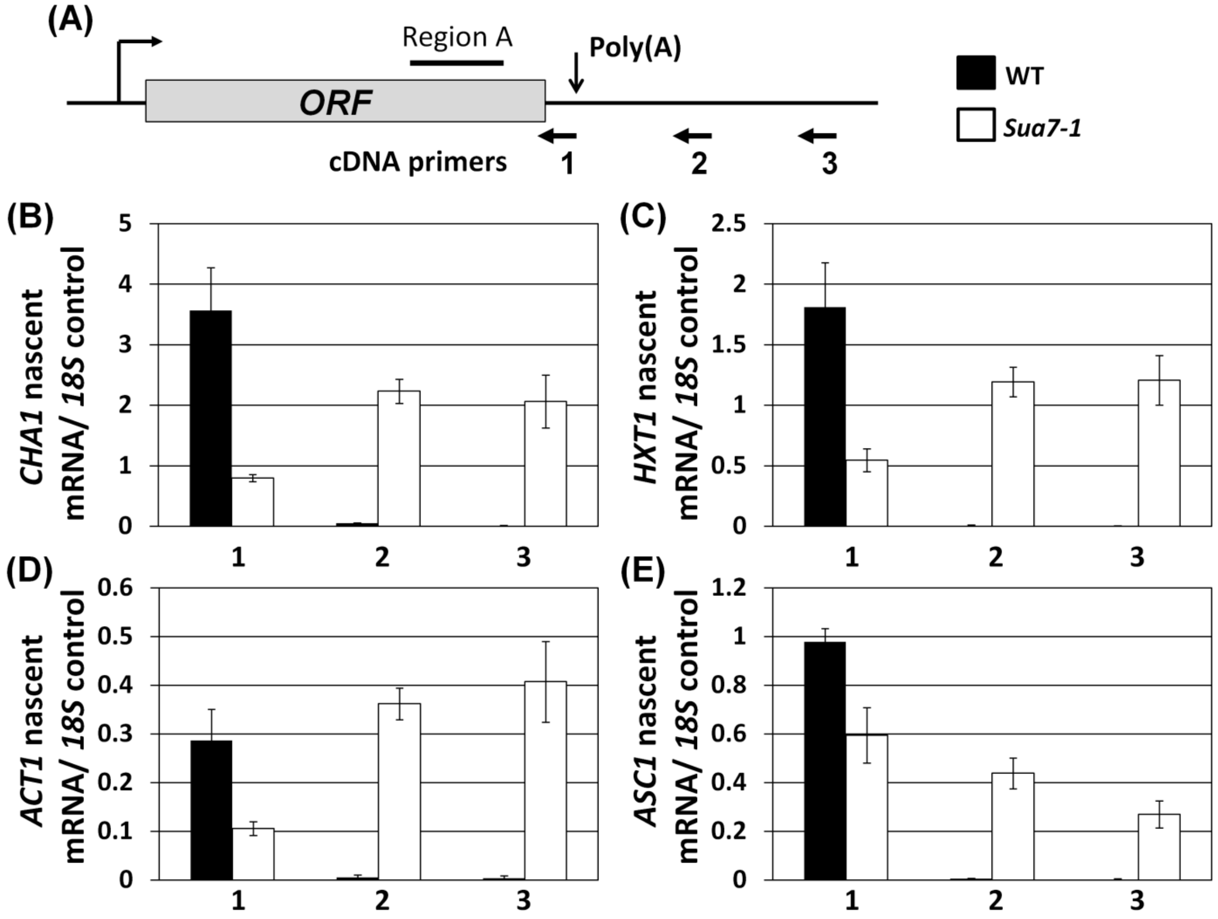

Figure S7. The looping defective mutant of TFIIB, *sua7-1*, exhibits termination defect. A, Schematic depiction of a gene showing the positions of three primers 1, 2 and 3 used for cDNA synthesis following TRO procedure. Region A represents the sequence amplified by PCR following cDNA synthesis. B – E, Quantification of RNA levels detected following TRO analysis of indicated genes in *sua7-1* mutant and the isogenic wild type strain. The transcript level of *18S* was used as the control for normalization of results. Error bars represent one standard deviation. The results shown here are averages of at least four PCRs from two independent RNA preparations.

Supplemental Fig. S8

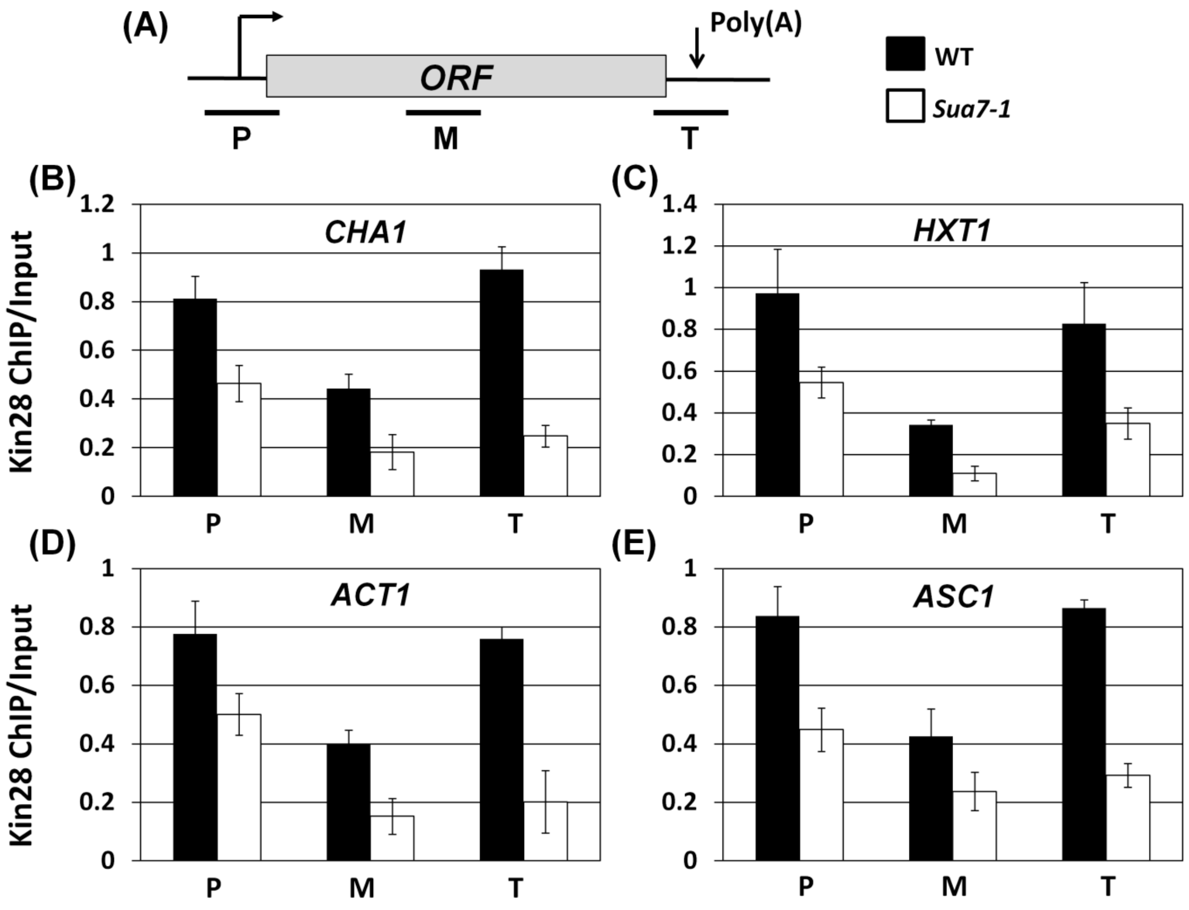

Figure S8. The looping defective mutant of TFIIB, *sua7-1*, exhibits a loss of Kin28 on the terminator. A, Schematic depiction of a gene indicating the position of the promoter (*P*), coding (*M*) and terminator (*T*) regions amplified by PCR in the ChIP assay. B – E, Quantification of ChIP signals showing crosslinking of Kin28 to different regions in wild type (WT) and mutant TFIIB (*sua7-1*) cells. Input represents DNA prior to immunoprecipitation. Error bars represent one unit of standard deviation. The results shown here are averages of at least four PCR reactions from two independent chromatin preparations.

Supplemental Fig. S9

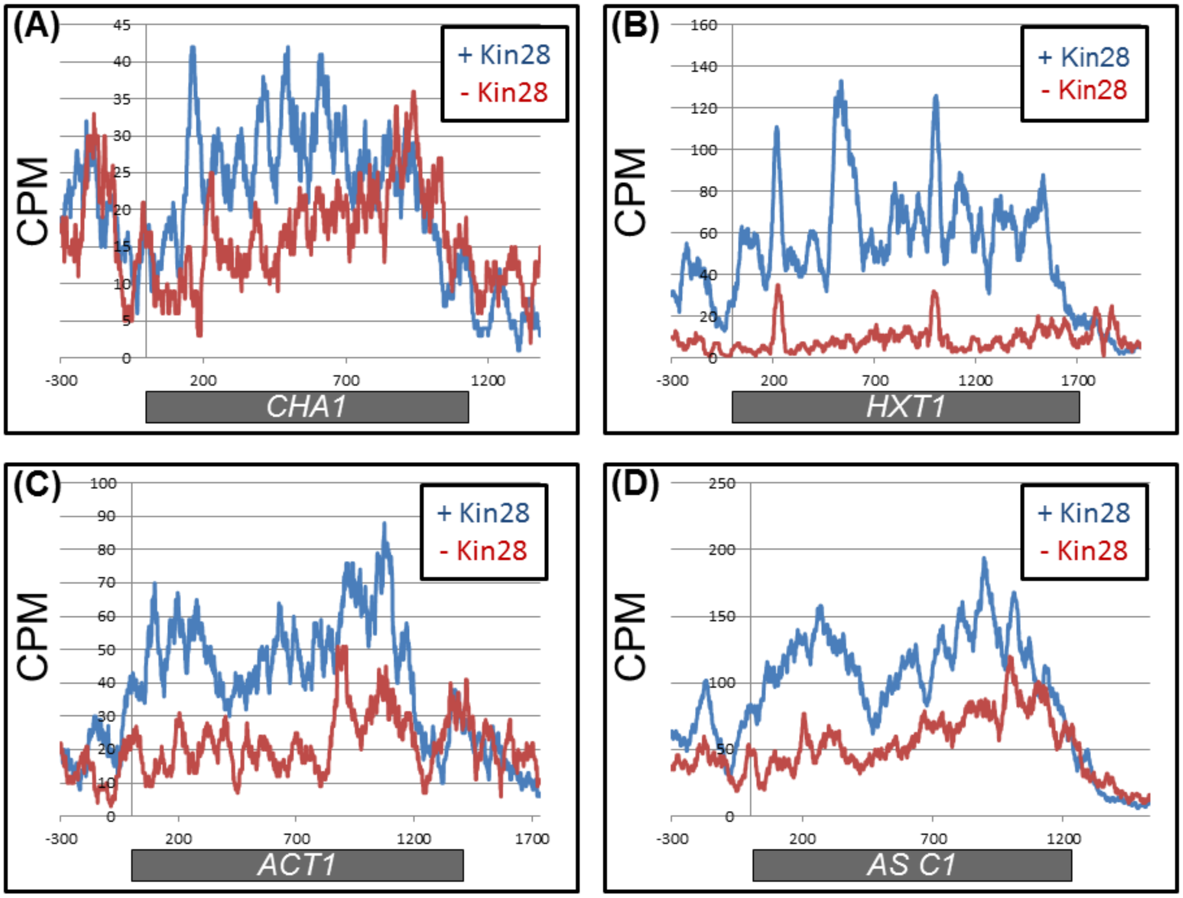

Figure S9. Genomewide RNAPII ChIP-Seq analysis performed by Kevin Struhl's laboratory showed a decrease in the polymerase density in the coding region and an increase in the polymerase ChIP signal beyond the 3' end of *CHA1, HXT1, ACT1* and *ASC1* in the absence of Kin28 kinase activity. RNAPII-density profile for *CHA1, HXT1, ACT1* and *ASC1* was extracted from the genomewide RNAP II ChIP-Seq data generated by Kevin Struhl's laboratory in the presence of Kin28 kinase (+Kin28) and upon depletion of Kin28 (-Kin28) from the nucleus by anchor away approach (Wong et al., 2014). The polymerase density profile for the four genes shown above is for the region encompassing the open reading frame (ORF), 300 bp upstream of ORF, and 300 bp downstream of the ORF. CPM is counts per million.

Supplemental Fig. S10

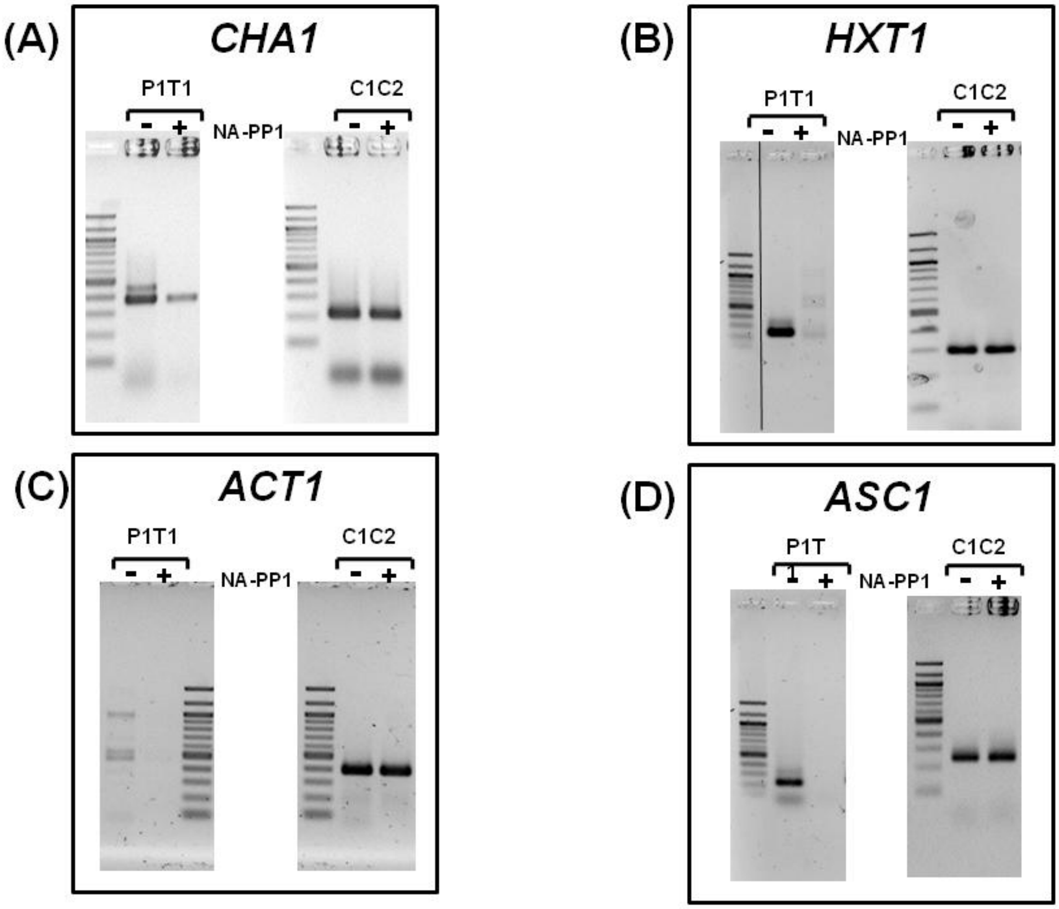

Figure S10. Full scan of gels shown in Fig. 5
